# Thou Shalt Be Reproducible! A Technology Perspective

**DOI:** 10.3389/fpsyg.2016.01079

**Published:** 2016-07-14

**Authors:** Patrick Mair

**Affiliations:** Department of Psychology, Harvard UniversityCambridge, MA, USA

**Keywords:** reproducibility, data archiving, R, LAT_E_X, knitr, R Markdown, open source

## Abstract

This article elaborates on reproducibility in psychology from a technological viewpoint. Modern open source computational environments are shown and explained that foster reproducibility throughout the whole research life cycle, and to which emerging psychology researchers should be sensitized, are shown and explained. First, data archiving platforms that make datasets publicly available are presented. Second, R is advocated as the data-analytic *lingua franca* in psychology for achieving reproducible statistical analysis. Third, dynamic report generation environments for writing reproducible manuscripts that integrate text, data analysis, and statistical outputs such as figures and tables in a single document are described. Supplementary materials are provided in order to get the reader started with these technologies.

## 1. Introduction

The field of psychology has been in an uproar in recent years over the replication/publication crisis, as researchers have been unable to replicate the results of (published) experiments (see e.g., Pashler and Wagenmakers, 2012). Replicating an experiment is a critical aspect of scientific practice, because it allows the reliability and repeatability of an experiment to be assessed.

There is some confusion in the literature regarding the distinction between *replicability* and *reproducibility*, and sometimes they are used interchangeably^1^. For this article it is imperative to clearly distinguish between the two. As pointed out in the preface of Stodden et al. (2014), *replication* is the practice of independently implementing scientific experiments to validate specific findings. It refers to the process of repeating the experiment by collecting new data (in exactly in the same way as in the original experiment), performing the same statistical analysis, and reaching the same conclusions.

A study is *reproducible* if there is a specific set of computational functions/analyses that allow independent scientists to exactly reproduce all the results obtained in the original study (Irizarry et al., 2012). According to Peng et al. (2006), reproducibility calls for data sets and software to be made available for (1) verifying published findings, (2) conducting alternative analyses of the same data, (3) eliminating uninformed criticisms that do not stand up to existing data, and (4) expediting the interchange of ideas among investigators. Thus, reproducibility can be thought of as a different standard of validity because it forgoes independent data collection and uses the methods and data collected by the original investigator.

We see that the core of this reproducibility definition is “reproducible data analysis”. This is the key component. However, as we will explore in the section on dynamic report generation, reproducible data analysis can be embedded into manuscript generation, which leads to fully reproducible manuscripts. Several articles have carried this idea into various fields: statistics (Gentleman and Temple Lang, 2007), biostatistics (Peng, 2009), econometrics (Koenker and Zeileis, 2009), and geosciences (Pebesma et al., 2012).

In this article we present technological tools, environments, and infrastructures that foster reproducible research at various stages. First, we can think of reproducibility in the actual research stage, that is, reproducible data preparation, statistical analysis, and documentation. Second, at the publication level, we can think of reproducible results for the reviewers (the mantra “trust but verify” by Simons, 2014 plays a key role here). Third, once the article is published, the findings should be reproducible by the readers, and give them the option to perform alternative analyses using the original data. How urgent it is for journals to make published research reproducible was pointed out in McNutt (2014): “A transparent and rigorous approach, however, can almost always shine a light on issues of reproducibility. This light ensures that science moves forward, through independent verification as well as the course corrections that come from refutations and the objective examination of the resulting data.” More generally, Gandrud (2014) adds that using reproducible research tools leads to better work habits, better teamwork, and higher research impact.

We focus on the following domains: data storage and archiving, a computational statistical environment for writing reproducible code, and dynamic report generation that combines the syntax from the statistical analysis with word processing. We limit our explanations to open source solutions that run on all major operating systems such that *every* researcher can reproduce analyses without having to purchase proprietary software, which is often limited to an exclusive set of institutions due to hefty licensing fees. The tools and infrastructures we are going to present are the Dataverse Network for data archiving (King, 2007), R (R Core Team, 2016) for reproducible statistical analysis, LAT_E_X (Lambert, 1994) and R Markdown (RStudio Team, 2016) in conjunction with an R package called knitr (Xie, 2013) for dynamic report generation^2^. Using these tools allows researchers in psychology to make their analyses, research reports, and publications fully reproducible.

## 2. Data archiving

### 2.1. Making datasets publicly available

Let us plunge into the very foundation of reproducibility: making datasets publicly available. If we want to reproduce any statistical analysis, we need to have access to the data. Unfortunately, the number of published studies in psychology that share the data publicly is still very low. Wicherts (2011) and Wicherts and Bakker (2012) elaborate in great detail on the importance of data sharing in psychology. Why is it that psychologists are reluctant to share their data?

Wicherts et al. (2011) examined the hypothesis that authors fear that re-analysis may expose errors in their work or may produce conclusions that contradict their own. They studied whether the researchers' willingness to share data is associated with two measures: (a) weaker evidence (against the null hypothesis of no effect), and (b) the quality of the reporting of statistical results (defined in terms of the prevalence of inconsistencies in reported statistical results). Regarding (a) they found that the reluctance to share data is associated with weaker evidence, and regarding (b) they found that reluctance to share data is associated with a higher prevalence of apparent errors in reported statistical results.

Apart from these empirical findings, researchers may also raise the following, more subjective objections to sharing data:

1. Institutional Review Board (IRB)/Ethics Committee concerns: If the IRB does not give the approval to share the data at all, there is nothing we can do about it. The IRB typically has fewer concerns if the data are sufficiently anonymized or de-identified, however, the concern can be raised that supposedly anonymous data could be re-identified. Researchers need to think through this issue carefully. In addition, some countries have more stringent IRB rules than others, but we nevertheless urge researchers to make an effort to find out whether it is possible to share their data on an anonymized level within their research context.
2. “I want to first fully analyze my data before someone else gets to it”: Undoubtedly, researchers must often go through considerable financial effort to collect data for some kinds of studies (e.g., in fMRI) and it is understandable that researchers would like to publish potentially multiple articles before sharing the data. Under such circumstances an option would be to share at least “sufficient statistics,” that is, measures such as (group) means, variances, correlation, and covariance matrices, etc. Sharing at this level would be helpful for meta-analyses as well as for some linear model based techniques. Once a dataset is fully squeezed out, it would be vastly helpful to the psychology community to share the data.
3. Fear that someone will engage in “*p*-bashing”: The term “*p*-bashing” is essentially the opposite of “*p*-hacking,” that is, finding a way to make one's results go away by, say, removing a single outlier.
4. “I never thought about it and I also wouldn't know where to upload the data”: This is a very common statement. In the past, psychologists were not too concerned about the issue of open science/reproducibility. However, since this is changing, let us elaborate on the “where to upload the data” part of the statement.

We can think of the following levels of data sharing: sharing data upon request, putting data on a personal website, submitting data to the journal website, and uploading data in a public data repository.

Sharing the data upon request is certainly not the standard we should aim for. Making the datasets publicly available on a personal website is an improvement, but with several problems: links are highly dynamic and impermanent, and there are no standardized regulations governing data storage (e.g., data could be stored in proprietary formats, contain no variable description, collection protocol, or any other metadata information, thereby making the raw data almost useless). If journals required authors to submit the data and provide corresponding storage infrastructures, that would be great—but then one needs to have journal access in order to use the data. This brings us to data sharing through open repositories, freely accessible to everybody: this is the gold standard we should aim for.

### 2.2. Dataverse project

Let us briefly review the “three pillars of data publishing” (Crosas and King, 2015) in order to make data in open repositories accessible and reusable:

1. Trusted data repository: to host and access data.
2. Data citation: to find and reference data.
3. Information about the data (metadata): to understand and reuse data.

The first pillar is merely technical. It implies, among other things, that the provider ensures long-term persistence and preservation of datasets in their published form and provides stable identifiers for submitted datasets. The second pillar simply means that researchers should get credit for sharing the datasets. Finally, without metadata (the third pillar), a dataset is pretty much useless. Metadata are the descriptions of the data such as variable names, factor levels, contextual background (e.g., how the dataset was collected), etc. An infrastructure that builds on these pillars is the Dataverse Project (see http://dataverse.org/).

The Dataverse Project is hosted by the Institute for Quantitative Social Sciences (IQSS) at Harvard University. It is an open source web application to share, preserve, cite, explore, and analyze research data. It facilitates making data available to others, and allows the user to reproduce others' work. Researchers, data authors, publishers, data distributors, and affiliated institutions all receive appropriate credit. Each Dataverse repository can contain multiple datasets, and each dataset contains metadata and related files such as documentation and supplementary syntax files.

Metadata are descriptions of the data, and Dataverse allows one to specify metadata at different levels (see Crosas, 2016). For reproducibility, file-level metadata are most important. At this level, metadata contain information about each variable in the dataset; in other words, researchers must be able to understand what variable names mean. This is the minimum requirement for reproducibility. For replicability, metadata need to be richer, and more detailed information needs to be provided concerning the study design, participant recruitment, materials/instruments/methods, and how the field values (variables) were derived. An international metadata standard for social science data is defined by the Data Documentation Initiative (DDI; see http://www.ddialliance.org/).

In order to submit a study to the Dataverse network, researchers can go to http://dataverse.org/, create an account, create a Dataverse, and upload their datasets, including metadata. Once the Dataverse is publicly released, anyone can download the dataset(s) in multiple formats, depending on the statistical environment the researcher is working in. There are several nice additional features. One is that the popular Open Science Framework (OSF; Open Science Collaboration, 2012) platform (see https://osf.io/) designed for collaborative research in psychology provides an interface to Dataverse. Another feature of Dataverse in relation to R (described in the next section), is the **dataverse** package (Leeper, 2016, https://github.com/IQSS/dataverse-client-r) which allows a researcher to manage his or her personal Dataverse from within R through Dataverse's powerful application programming interface (API; see http://guides.dataverse.org/en/4.2/api/). This API allows other developers to build tools which interoperate with Dataverse.

A final, practical concern relates to the research stage at which a dataset should be uploaded to Dataverse and publicly released. A Dataverse can be created as soon as data collection is complete. As long as one does not click on the “publish” button, the dataset is not visible to the public but can be shared among the member of the research team. Corresponding code files for the statistical analysis can be uploaded as well. At that stage the Dataverse receives a DOI (digital object identifier), which is a serial code used to uniquely identify objects such as publications and data. This makes the dataset citable, if needed. During the article review process additional updates to the Dataverse may be made. A Dataverse should be published once the data and the code are in a presentable shape, which may be tied to the acceptance of an article.

An example of the correct way to cite a dataset on Dataverse if the data are to be used for further investigation is the following:

Mair, P., Hofmann, E., Gruber, K., Hatzinger, R., Zeileis, A., and Hornik, K. (2016). “Replication Data for CRAN study,” http://dx.doi.org/10.7910/DVN/7EKMC8, Harvard Dataverse, V1.

## 3. Statistics! R in psychology

### 3.1. Why use R?

The answer to this question is that R is the most comprehensive statistical software product on the market, its syntax leads to reproducible statistical analyses, and, last but not least, it is freely available. Since it was initially proposed by Ihaka and Gentleman (1996), R has had a meteoric rise into the echelon of statistical computing environments. How did that happen and what is the benefit of using R in psychology?

As pointed out already in the Introduction, state-of-the-art designs for psychological experiments have become increasingly complex and statistical applications in the field are extremely broad: on one end of the spectrum we can think of personality psychologists who often use latent variable techniques or social psychologists applying ANOVA techniques; on the other end we have researchers in the fMRI area who use approaches from machine learning on four-dimensional data structures.

An illustrative scenario of a modern experiment is that researchers have behavioral data from classical questionnaires and want to combine them with fMRI data where each participant, while being scanned, has to perform multiple tasks. The analysis of the behavioral data can involve scaling methods such as item response theory (IRT) models or structural equation models (SEM). On the neuroimaging side, the analysis might require support vector machines (SVM), multi-voxel pattern analysis, or Bayesian approaches. Since in fMRI experiments having a large sample size is often too cost-intensive, inferential statistics might also involve permutation tests. When examining relationships between the behavioral responses and the beta values from the brain scan, mixed-effects models might be applied due to the within-between subjects nature of the data. In addition, the researcher might want to visualize relationships between the variables by applying multivariate exploratory methods such as multidimensional scaling (MDS). In the past, researchers had to obtain and learn several different (proprietary) statistical environments in order to perform such a comprehensive analysis.

“Can we do all these computations in R?” Yes! In R, all these methods are integrated in a single computational environment. The amount of statistical methodology implemented in R is vast. There are thousands of programmers around the world from many different fields including psychology who develop packages (see Mair et al., 2015, for a recent empirical study) and make them freely available by disseminating them through the official CRAN (Comprehensive R Archive Network) website. From a user's perspective there are basically no computational and methodological limits to the creativity of statistical analysis.

### 3.2. Understanding the R environment

R can be thought of as a Lego system. First, one can go to the CRAN website (http://cran.r-project.org/) and download the base R system. Since R is a script language, commands must be written as code in an editor—there's no clicking! RStudio (http://www.rstudio.com/) is the most popular one these days which provides a user-friendly working platform. R and RStudio also run on multiple operating systems: it does not matter if one is using Windows, Mac OS, or, more adventurously, Linux. In addition, since R is open source, there is no need to buy a license key and have it renewed every year.

Base R comes with basic statistical methodology. It can perform *t*-tests, χ^2^-tests, ANOVA, regression, etc. It also has pretty sophisticated data visualization capacities, a huge selling point of R. The Lego aspect comes in when special methodology is needed. For instance, SEM are not included in base R. But this is not a big deal: one can just “attach” a corresponding package to base R to fit an SEM. The same applies to SVM and friends; there is pretty much a package for everything. There are several thousands of add-on packages available on CRAN, with more coming in every day. R packages are implemented in a standardized way which makes it fairly easy for someone with basic R knowledge to explore and use a new package.

### 3.3. On learning R

Learning R means learning a script language. This can be challenging if someone only been previously exposed to a GUI (graphical user interface) based software such as SPSS (IBM Corp., 2015): there is no ANOVA button. What separates R syntax from SPSS syntax is that R is an actual programming language that is based on the mantra “everything in R is an object.” This implies that all modeling components throughout the analysis work flow (e.g., data subsets, variable transformations, model fit, parameters) can be stored as objects and re-accessed later for further computation. This concept leads to a perfectly reproducible data analysis.

At the beginning some basic concepts need to be learned and digested before one can actually perform any statistical analysis. These include calling functions with a proper argument specification, storing the results of functions as objects, and extracting various elements of these objects. At the beginning of a students' R career, there is typically a certain level of frustration when he or she repeatedly gets these cryptic, and (even worse) bright-red error messages on the screen which do not seem to specify what is not working. To learn R requires patience and practice.

Once having this conceptual understanding of how the R syntax works, things become more intuitive and the statistical power and flexibility of R starts to open up. There are basically no limits in statistical analysis (especially by making use of the countless supporting resources available online) and one can become very creative with both exploratory data analysis and statistical modeling.

First year graduate students might think that it is too difficult to learn both statistics *and* R at the same time: It must be like climbing two 8000 meter peaks at once. In fact, one can beautifully learn statistics *through* R at a basic level as well as at a more advanced statistical modeling level.

At such an advanced modeling level (involving mixed-effects models, nonlinear regression techniques, Bayesian inference, etc.), the use of R has an additional benefit: one actually needs to know what he or she is doing in order to fit a statistical model. In our opinion, it is essential that researchers have a genuine statistical proficiency and training in order to be able to fit such models, required by complex data settings. When using GUI based software, students tend to try out different buttons (the method itself is pretty much a black box), and when they have a result that seems reasonable, they approach the quantitative section of the department and ask: “How can I interpret this model?” In R, however, even at the early stage of model specification—and hopefully after making use of R's amazing capabilities to visualize their data—students need to know precisely what they are doing. They will not go too far with a trial-and-error strategy.

Learning R is hard work at the beginning, but it definitely pays off at the end; especially with respect to a graduate student's future career as a researcher. Some learning resources are given in the discussion section.

### 3.4. R and reproducible research

Research in psychology is typically a collaborative effort: sometimes different team or lab members contribute to the statistical analysis. Everyone should have the “big picture” of the analysis work flow so they have the opportunity to catch possible flaws in the analysis or provide improvements. This can not be achieved if the statistical analysis is performed through a GUI where ANOVA tables and various plots are emailed back and forth between the team members. Using a statistical script language such as R, the whole analysis work flow is shared among the team members such that the analysis is reproducible at each point in time during research, instead of just sharing only outputs. This makes the data analysis process highly efficient (different team members can contribute different code chunks), transparent (each one has full insight into the analysis), and, most important, reproducible (each team member can fully reproduce the analysis).

Once an article has been published, readers want to learn from it. This can be difficult if it is not clear exactly how the analysis described in the article was performed. For example, without having the underlying script nor the data available it will forever remain a mystery why the authors used that fancy Goodman correlation coefficient instead of a simple Pearson correlation. Even in the instance where the statistical part of the paper is flawless, for a reader with a similar data setup it would be a tremendous asset to be able to reproduce the original article and adapt the computation to their own data.

Last but not least, it is worthwhile to mention that R also has an indirect effect on replicability. Above we mentioned how important it is for researchers in our field to have a profound understanding of statistical inference. In relation to replicability, we can determine at least three key elements that produce the replication crisis: First, psychologists often do not have a good understanding of statistical power and typically use null hypothesis significance testing. As Maxwell et al. (2015) point out, solutions to these problems include meta-analysis and Bayesian approaches for which R provides a large amount packages. Second, researchers do not make a clear distinction between a priori model testing, exploratory *post-hoc* model modification, and exploratory model development (Diaconis, 1985). Third, researchers engage in procedures that increase the Type I error rate (discarding conditions, data, altering measures, etc.) in search of statistical significance (Simmons et al., 2011). An environment like R provides the researcher with tools to get a detailed insight into these concepts through simulation studies and corresponding visualizations.

## 4. Generating dynamic reports

Let's face it: The vast majority of psychology articles are written in Microsoft (MS) Word—and this will not change in the near future. The only bizarre psychological subculture that uses something different (to be more precise, a word processing tool that goes by the kinky name of LAT_E_X), are psychometricians. We are not here to say whether MS Word is good or bad. Nevertheless, in this section we introduce open source alternatives that allow us to generate dynamic reports and may have an impact in the future.

Let us start with a simple example which shows what dynamic reports are doing for us. Assume that the R manifesto above was convincing to the reader, and he or she was able to assemble an R code file that provides a fully reproducible analysis to all research team members. Now the graduate student starts writing a paper in MS Word which, as statistical output, contains a scatterplot with abbreviated variable names on the axes and a regression table containing the parameter estimates, the Wald statistic, and the *p*-values.

The advisor tells the student that he or she should use the full variable names for the scatterplot axes and put the standard errors of the regression parameters in the table as well. Doing this in a “standard” way would imply to go back to the R code chunk. First, the axes labels when calling the scatterplot function need to be modified. This plot needs to be save and then imported into MS Word. This also involves that the plot has to be resized and put where it should be in the document. Second, the regression needs to be recomputed and the table recreated in Word with a new column containing the standard errors through copy-paste. Again, the table needs to be properly resized, and its style needs to obey the journal guidelines.

As we see there are many non-reproducible copy-paste steps involved. Wouldn't it be great if we were to have a word processing tool that is able to combine the text in the paper with the R code that directly produces the output one wants to show in the article? In other words, one makes the (tiny) R code changes directly in the document (i.e., axes labels, standard errors) and, after running a command, the new plot and the new table are immediately and flawlessly integrated into the paper.

This concept is called *dynamic report generation* and has many advantages. Changes in the data (e.g., removing an outlier) lead to immediate changes of all the statistical outputs in the article. Changes in the analysis (e.g., changing a parameter restriction in a complex SEM) leads to immediate changes of the outputs in the article. The issue associated with reporting errors such as inconsistent *p*-values (see Nuijten et al., 2015) is minimized. When working with dynamic reports, it is easy to pull out the code upon which the analysis is based; this way, statistical outputs in the manuscript may be reproduced exactly (sometimes supplementary code is submitted which does not produce exactly the same outcomes as in the paper). Documents can be compiled into various output formats such as pdf, docx, and HTML. In relation to reproducibility and transparency, as pointed out in the Introduction, Gandrud (2014) claims that dynamic documents lead to better work habits (e.g., a clear, dynamic documentation helps one to avoid errors or makes it easier to find errors) and better teamwork (e.g., it is easier for the collaborators to understand the analysis in the article).

Below we are going to present two tools for dynamic report generation. The first tool is the combination of LAT_E_X and R code via a package called **knitr** which can be used for producing a journal article which needs to obey a particular formatting style. A second tool, super easy to use is R Markdown which allows one to create dynamic reports issued as HTML, docx, or pdf.

### 4.1. The LAT_E_X markup language

We have heard the name LAT_E_X already. First of all, LAT_E_X people hate if LAT_E_X is called a “word processing tool,” as we shamelessly did above. We do not want that LAT_E_X people hate us; therefore, from now on, we use the proper term “markup language.”

Prima facie, LAT_E_X has nothing to do with R. As a markup language, LAT_E_X is a system for annotating a document in a way that is syntactically distinguishable from the text. This means that LAT_E_X documents can have environments like figures, tables, sections, and formulas. In the text these objects are referenced. As an example, if figures in a text have to be reordered, LAT_E_X immediately reassigns the figure numbers properly. The same applies to sections, tables, etc.

That's all good news. The bad news is that writing in LAT_E_X is not that easy, especially if someone has been primed since early childhood with MS Word. Loosely speaking, LAT_E_X relates to MS Word as R does to SPSS. The most difficult issue for beginners is typically that LAT_E_X has no WYSIWYG (“What You See Is What You Get”). For instance, if one makes a word bold in MS Word, one immediately sees the bold word on the screen. In LAT_E_X, there is a “command” (called environment) for making a word bold. The researcher works in a script file and only when compiling, that is, converting the script into a pdf (portable document file), one sees the resulting bold word on the screen in a pdf viewer.

Why then are psychometricians actually using LAT_E_X if things seem to be so cumbersome? Journals in this field such as *Psychometrika* provide LAT_E_X style files for download. By calling this style file in your LAT_E_X script, the resulting pdf file corresponds exactly to the style formats required by the journal—including the bibliography style. Therefore, in LAT_E_X one never has to worry about style issues. In addition, there is one more aspect which is of a purely aesthetic nature: LAT_E_X documents just look beautiful! Tables, figures, and formulas (which psychometricians tend to use a lot) are seamlessly integrated into the document.

Apart from the psychometrics community there are several journals relevant to the wider population of psychological researchers that provide LAT_E_X style files and templates. Some examples are: Frontiers series, PNAS, PLoS ONE, Science, and Nature. Many other psychology journals, however, require APA style (American Psychological Association, 2009). To prepare a LAT_E_X document in APA style, one can simply “load” the **apa6** package (Beitzel, 2013) and the corresponding citation style implemented in **apacite** (Meijer, 2013). As we see, LAT_E_X has the same Lego concept as R. Needless to say that LAT_E_X is open source and runs on multiple operating systems, just like R.

### 4.2. Marrying R and LAT_E_X: the knitr package

So far we have seen how to reproduce a statistical analysis using R. Then we talked about LAT_E_X as an environment where we write our manuscript. However, we are still at a level where we have to insert numerical results and figures from R into our tex-file manually.

Now we get nerdy and try to integrate R code into a LAT_E_X document such that when the LAT_E_X file is compiled, the full analysis is computed “on-the-fly” and the desired outputs (descriptive measures, tables, plots, etc.) are then integrated into the pdf. In order to marry LAT_E_X with R, Leisch (2003) developed the **Sweave** package for R. More recently, Xie (2013) developed the **knitr** package which solves some problems **Sweave** had and combines features of other add-on packages.

Using **knitr**, the report is automatically updated if the data or some steps of the analysis change. As mentioned above, we want to avoid inserting a pre-fabricated graph, table, or any other statistical outputs into the report. Using the **knitr** package, this process is called “knitting.” An example is given in the Supplementary Materials.

### 4.3. R Markdown

Another recent tool for dynamic document generation is R Markdown (RStudio Team, 2016). It is designed specifically for dynamic reports where the analysis is carried out in R and it provides an incredible amount of flexibility. On the syntax side it uses Markdown which is a very simple to use markup language, much easier than LAT_E_X.

Internally it builds on **knitr** such that all the benefits of dynamic reporting mentioned above apply to R Markdown as well. That is, it offers a seamless integration of R code and text. The output format does not necessarily have to be a pdf, it can be HTML (e.g., for online versions of articles), but also MS Word—which makes it interesting for psychologists who shy away from LAT_E_X. A simple example is given in the Supplementary Materials whereas Gandrud (2014) gives more detailed explanations.

## 5. Summary and discussion

This article presented several technological tools that ensure reproducibility at various stages of the research life cycle. We have focused on data archiving (Dataverse project), statistical analysis (R environment), and dynamic report generation (**knitr** package in conjunction LAT_E_X markup or R Markdown). We did not address the topic of collaborative research, which is an important issue within the reproducibility and replicability discussion, as it has already been covered in detail in the article by Open Science Collaboration (2012). We suggest that the reader check out the OSF platform, as it nicely interacts with the tools presented here.

All the technologies presented in this article are open source. Open source products have been suffering from “if it's free, it can't be good” prejudices, and have earned many snide criticisms from proprietary software companies and researchers. The tools discussed here are, however, leading in their respective niches. For instance, in the case of R, there is no proprietary product on the market that has close to the same overall capabilities in terms of the variety of statistical models. Additionally, the stellar impact of these open source environments is tied to the democratization of research. Reproducibility should not be limited to researchers in elite institutions, but should rather be doable for researchers at universities in less-developed countries as well. The technologies presented in this article give researchers in all countries the ability to contribute to the research community at a highest level. If the reader is interested in additional compelling, and somewhat more aggressive arguments for the necessity of free software, we suggest that they look at the GNU manifesto by Stallman (1985).

Each technology has its limitations. In the case of R there are still some domains where other environments provide more comprehensive implementations. For example, MATLAB (The MathWorks, Inc., 2016) has a strong tradition in the cognitive neuroscience area where it is still the leading computational environment (e.g., it offers better pre-processing functionalities than R). However, R is gaining ground in cognitive neuroscience; Eloyan et al. (2014) give a tutorial on how to perform fMRI analysis in R. Another example is **lavaan** (Rosseel, 2012) vs. MPlus (Muthén and Muthén, 2015) for path analysis and latent variable modeling. Mplus has still some features like multilevel SEM and mixture SEM not yet offered by **lavaan**. However, there is also the **OpenMx** (Neale et al., 2016) package in R which allows for more advanced SEM specifications.

R is not the most efficient platform for processing massive datasets, a scenario which psychologists are not likely to encounter (except for some large scale fMRI settings). In this case Python (Python Software Foundation, 2016) is a good open source alternative. It is extremely popular in the field of Data Science where large amounts of data have to be analyzed. It can be expected that in the future, especially in the fMRI area, Python will have an impact. Similar to **knitr**, the **knitpy** package provides a powerful environment for dynamic report generation.

What will be the impact of R in psychology in the future? Making this type of prediction is always difficult. In recent years R has already “invaded” many applied fields such as ecology, biology, health sciences, political sciences, etc. In Psychology, an increasing number of lecturers are teaching R in graduate and even undergraduate courses, and more and more researchers have started using R. It will be interesting to observe the increase of R applications in psychology of the next years.

When using LAT_E_X for APA style documents, the **apa6** package provides an almost perfect automatization. This means that there are currently some idiosyncratic formatting requirement which **apa6** does not get completely right, and it can be tedious to get that last bit of customization perfect.

As far as dynamic report generation is concerned, there are a few things that should be addressed. For complex models which take a long time to estimate [such as a Markov Chain Monte Carlo (MCMC) simulation in a Bayesian model], it is annoying to wait for several minutes (or hours) until the MCMC simulation is finished each time we compile the document. The **knitr** package provides an option for caching the results. This means that the computation is only performed once and the results are stored. As long as no code changes are made in the respective MCMC chunk, the stored results are used for each further compilation.

Dynamic documents allow the reader to create all statistical outputs in the manuscript “on-the-fly.” Statistical outputs are presented by means of tables, figures, but also in-line numbers in the results (**knitr** provides the Sexpr command for this; an illustration is given in the Supplementary Materials). Let us say we remove some outliers from the data, which leads to changes in the parameter estimates, standard errors, plots, etc. Correspondingly, the interpretation may change. Using dynamic document generation all the outputs in the manuscript are immediately changed. While this is clearly very elegant, there is also some danger in it. The user needs to make sure that he or she is not losing track of these changes and is properly adapting the interpretation in the corresponding text. An alternative hybrid approach, if one does not want to be completely orthodox about fully reproducible dynamic documents, is to create a document in which only some of the R outputs are dynamic, while others are “hard coded” in the document.

Instead of lamenting the steep learning curves of some of the technologies presented in this article, here are a few resources the reader can consider in order get acquainted with them. As far as R is concerned, a great platform is the R Data Camp (https://www.datacamp.com/), where the user gets gently introduced to R by means of several motivating modules. In terms of books, “Learn R in a Day” (Murray, 2013), “R for Dummies” (de Vries and Meys, 2012), and the “R Cookbook” (Teetor, 2011) give the reader a very easy start. In the “R Graphics Cookbook” (Chang, 2012) the reader finds detailed information on how to produce fancy plots. If more statistical substance is needed, the entertaining “Discovering Statistics Using R” book by Field et al. (2012) is one of the best quantitative books for psychologists on the market. Once done with the basics, http://rseek.org/ can be used as an R search engine. For those who want to be up-to-date on the latest R developments and discussions, http://www.r-bloggers.com/ is a great resource.

In terms of LAT_E_X, the components needed to run LAT_E_X are described in the Appendix in Supplementary Material. RStudio can be used as an editor. If someone only wants to invest a few minutes learning it, one can watch the (6 min long) “Learn LAT_E_X in 5 Minutes” YouTube tutorial video (https://www.youtube.com/watch?v=Y-kXtWdjtmw). If someone can spare a bit more time, a well-structured Wikibook on the LAT_E_X basics can be found at https://en.wikibooks.org/wiki/LaTeX/Basics. Sasha Eskamp (http://sachaepskamp.com/?p=232) provides online access to the materials of his “LAT_E_X for psychology researchers” seminar.

Tables are a special challenge in LAT_E_X. A Wikibook (see https://en.wikibooks.org/wiki/LaTeX/Tables) provides details on how to produce simple as well as more complex and customized tables in LAT_E_X. An introduction for using LAT_E_X in APA style is provided by William Revelle, see http://www.personality-project.org/revelle/syllabi/205/apa.style.html.

The Supplementary Materials should give the user a good starting point for **knitr** in conjunction with LAT_E_X. If the reader is more attracted to R Markdown, the official website (http://rmarkdown.rstudio.com/) and the following R-blogger entry give good introductions: http://www.r-bloggers.com/getting-started-with-r-markdown-knitr-and-rstudio-0-96/. More depth is offered in the books by Xie (2013) and Gandrud (2014).

In conclusion, the reader is now equipped with enough great open source technologies and corresponding resources which make him or her dangerous. It is our hope that the article motivates the reader to delve deeper into these great (and sometimes admittedly nerdy) environments in order to make his or her research fully transparent and reproducible.

## Author contributions

The author confirms being the sole contributor of this work and approved it for publication.

### Conflict of interest statement

The author declares that the research was conducted in the absence of any commercial or financial relationships that could be construed as a potential conflict of interest.

## Footnotes

^1^This issue is excellently summarized in a post by Mark Liberman (see http://languagelog.ldc.upenn.edu/nll/?p=21956).

^2^Note that this list of tools is by no means exhaustive as there exist several other great environments.
