# Supplementary material for: Thou Shalt Be Reproducible! A Technology Perspective
Source: Front Psychol. 2016 Jul 14;7:1079. doi: 10.3389/fpsyg.2016.01079 (PMC4943952; doi:10.3389/fpsyg.2016.01079)
Supplement: Supplementary file 1 [file Instructions.pdf]

How to Work with the Supplementary Materials

The supplementary materials (zip-file) consist of four independent parts:

1. Reproducible data analysis using R.
2. Simple dynamic report written in R Markdown (and **knitr**).
3. Simple dynamic report generation written in LaTeX (and **knitr**).
4. Source files for two of my personal articles written in **knitr**.

Reproducible Data Analysis Using R

Assuming that R and RStudio are installed, open the file `RBasicsExample.R` in RStudio. This file shows how to fit basic statistical models such as *t*-test, two-way ANOVA, regression, and, slightly more advanced, a mixed-effects model (growth curve model). It also includes basic plotting strategies and some useful data manipulation commands. The user can go through the code line-by-line: `Ctrl+Enter` sends the corresponding code line in the editor panel down to the console where it is immediately executed.

Dynamic Report Generation I: R Markdown

R Markdown files are stored as `*.Rmd` files. They contain plain text, some special text syntax (e.g. for bold, italics, headers, etc.), and R code chunks. In the supplementary materials, two Rmd-files are provided. Let us start with `RMarkdownExample1.Rmd` by opening it in RStudio. This file contains a regression analysis and compiles the document as HTML file. All that needs to be done is to click on the "Knit HTML" button which should have appeared at the top of the editor window. The resulting `RMarkdownExample1.html` can be opened in a browser.

`RMarkdownExample2.Rmd` has the same content as `RMarkdownExample1.Rmd`, the only difference is that this time we compile it as Word file. After opening it in RStudio, a "Knit Word" button should appear. By clicking on it, `RMarkdownExample2.docx` will be created, that is, a dynamically produced Word document.

More details on the R Markdown syntax used in the Rmd-files can be found in the following cheat sheet: `https://www.rstudio.com/wp-content/uploads/2015/02/rmarkdown-cheatsheet.pdf`

Dynamic Report Generation II: LaTeX

Before we delve into the example file, a few general remarks. In order to work with LaTeX, the user needs to install either MiKTeX (`http://miktex.org/`; Windows only) or TeX Live (`https://www.tug.org/texlive/`; Windows, Mac OS, and Linux). A LaTeX document is typically stored as `*.tex` file, which is prepared in a LaTeX editor. RStudio can be used as LaTeX editor. The tex-file is then compiled into a pdf.

References are typically kept in an external bibliography file (bib-file) which contains templates for journal articles, books, edited volumes, unpublished articles, theses, etc. The bib-file itself is independent from the citation format and is included at the end of the tex-file. This produces the reference list in the corresponding style format required by the journal.

As supplementary materials we provide a simple example of a dynamic report in APA style which combines LaTeX code with R code. Three files are provided: an Rnw-file containing LaTeX and R code, a bib-file with the references, and the pdf output file.

The document includes various LaTeX environments often needed in manuscripts: bold fonts, italics, regular tables, dynamic statistical tables and figures, formulas, URLs, etc. as well as textual and parenthetical citations of books, articles, and manuals as defined in the bib-file.

In order to produce the dynamic document, R, RStudio, and LaTeX need to be installed. In R, the following packages need to be installed: **knitr**, **car**, **psych**, and **xtable**. The LaTeX packages **apa6** and **apacite**, which come with the full LaTeX installation, are required as well.

The file setup for knitting is the following. The master file has the ending `*.Rnw`. This file includes the LaTeX script and the R code. Whenever a chunk of R code appears in a certain part of the Rnw-file, this is marked with a special tag. The final pdf is now ctreated by the following two steps: a) performing the statistical computations in R and integrating the desired statistical output into a tex-file, and b) converting the tex-file into a pdf.

The **knitr** package provides the convenience function `knit2pdf()` in R which performs both steps at once. This implies that the user does not have to touch the tex-file at all: all the LaTeX and R components go into the Rnw-file and through knitting we get the pdf as output. The steps are the following:

1. Open the `KnitrExample.Rnw` file in RStudio. Make sure that R is in the directory where the Rnw-file is saved (see `setwd()` if the path needs to be changed).

2. Load the **knitr** package via `require(knitr)`.

3. Say `knit2pdf("KnitrExample.Rnw")` which produces the pdf output document.

If no major accident happened, the resulting pdf should look the same as the pdf provided in the Supplementary Materials. The Rnw-file includes many comments (lines that start with the percentage sign) which give detailed explanations. The bib-file, which can also be opened in RStudio, provides various templates for books, articles, and manuals.

Finally, by saying `purl("KnitrExample.Rnw")` in R, the R code from the paper is extracted and stored as `KnitrExample.R`. Such a code chunk can be submitted as an article supplement; this way, the reader can fully reproduce the analysis, assuming that the data are submitted as well or stored in a publicly accessible data archive.

Finally, here are a few details that we find helpful when we knit our documents. In the tags related to the R code chunk the user has many options: For instance, one can allow the R code to be visible or invisible in the pdf, one can force a plot to have a certain size as it should appear in the pdf, or one can

create and tailor a table directly through the R code chunk such that it appears properly formatted in the pdf. For the latter the **xtable** package does the job.

Dynamic Report Generation III: Example Articles

In the supplementary materials the reader can find two folders: CRAN and GOP. The CRAN folder contains the **knitr** source files including the journal style files, the dataset, and the bib-file of the article:

Mair et al. (2015). Motivation, values, and work design as drivers of participation in the R open source project for statistical computing. *PNAS, 112*, 14788-14792.

By saying `knit2pdf("cranpnas.Rnw")`, the article including all the analyses is fully reproduced as it was submitted to *PNAS*.

The second folder (GOP) contains the materials to reproduce the article:

Mair et al. (2014). The Grand Old Party: A party of values? *SpringerPlus, 3:697*

The command `knit2pdf("GOP.Rnw")` does the dynamic report generation job.

In both cases one needs to make sure that R is in the corresponding working directory and the packages loaded in the Rnw-files are installed. The datasets for both publications, including code that reproduces the analyses, can be found on Dataverse as well (search for "CRAN study" and "Republican statements" on `https://dataverse.harvard.edu/`).
