# Supplementary material for: Thou Shalt Be Reproducible! A Technology Perspective
Source: Front Psychol. 2016 Jul 14;7:1079. doi: 10.3389/fpsyg.2016.01079 (PMC4943952; doi:10.3389/fpsyg.2016.01079)
Supplement: Supplementary file 2 [file Reproducibility.zip › KnitrExample.pdf]

Personality Traits and Severity of Depression: A Regression Analysis

Larry Latex

Latex University

Kurt Knitr

Knitr University

Abstract

In this document we show how create a dynamic report using a simple linear regression as an example. On the LaTeX side it uses formula and figure environments. The whole statistical analysis (scatterplot, regression fit) written in `R` is embedded in the LaTeXscript. We use the `knitr` package for producing the pdf output.

Personality Traits and Severity of Depression: A Regression Analysis

The Linear Regression Model

Simple linear regression is one of the very basic techniques in statistics. The book by Fox and Weisberg (2011) provides a gentle introduction to various regression techniques.

A simple linear regression model can be formulated as $y_i = \beta_0 + \beta_1 x_i + \varepsilon_i$. In our sample we have n subjects ($i = 1, \ldots, n$) with predictor values x_i and response values y_i. The parameter β_0 is the intercept and the parameter β_1 is the slope. There is this other weird thing ε_i which is called "error term".

Regressing Depression on Personality Traits Using R

In this article we compute a simple linear regression example in `R` (R Core Team, 2015). `R` can be downloaded from `http://cran.r-project.org/`. We use a dataset from the `psych` package, written by Revelle (2015). This package includes a dataset with a Beck Depression Inventory measure (Beck, Ward, Mendelson, Mock, & Erbaugh, 1961), used as our dependent variable in the regression, and a neuroticism measure from the Eysenck Personality Inventory (H. J. Eysenck & Eysenck, 1975; S. B. G. Eysenck, Eysenck, & Barrett, 1985) as our independent variable. The traits associated with the three dimensions in Eysenck's personality model are given in Table 1.

[INSERT TABLE 1 HERE]

Results

Let us have a look at the dataset. The following code chunk shows the first 6 observations of our dataset.

```
require(psych)                              ## load the psych package
head(epi.bfi[, c("bdi", "epiNeur")])        ## first six observations

##   bdi epiNeur
## 1   1       9
## 2   7      12
## 3   4       5
## 4   8      15
## 5   8       2
## 6   5      15
```

In total, the dataset has 231 observations.

A fancy scatterplot function is provided by the `car` package. The scatterplot can be created using the following code chunk:

```
scatterplot(bdi ~ epiNeur, xlab = "EPI Neuroticism", ylab = "BDI",
            main = "Scatterplot", data = epi.bfi)
```

[INSERT FIGURE 1 HERE]

The scatterplot in Figure 1 shows somewhat of a linear relationship between the variables. The plot shows the regression line (green) as well as a nonparametric regression fit (red) including confidence intervals which show possible departures from linearity. Now we are ready to fit the linear regression model.

```
fitreg <- lm(bdi ~ epiNeur, data = epi.bfi)     ## regression fit
summary(fitreg)
```

```
##
## Call:
## lm(formula = bdi ~ epiNeur, data = epi.bfi)
##
## Residuals:
##      Min       1Q   Median       3Q      Max
## -11.9548  -3.1577  -0.7707   2.0452  16.4092
##
## Coefficients:
##             Estimate Std. Error t value Pr(>|t|)
## (Intercept) -0.32129    0.73070   -0.44    0.661
## epiNeur      0.68200    0.06353   10.74   <2e-16 ***
## ---
## Signif. codes:  0 '***' 0.001 '**' 0.01 '*' 0.05 '.' 0.1 ' ' 1
##
## Residual standard error: 4.721 on 229 degrees of freedom
## Multiple R-squared:  0.3348,Adjusted R-squared:  0.3319
## F-statistic: 115.3 on 1 and 229 DF,  p-value: < 2.2e-16
```

The output above shows the standard `R` output from the console. The same results in table format are given in Table 2.

We see that EPI has a **significant** impact on BDI. The 95% confidence interval for the slope parameter is [0.56, 0.81]. This concludes our little analysis.

References

Beck, A. T., Ward, C. H., Mendelson, M., Mock, J., & Erbaugh, J. (1961). An inventory for measuring depression. *Archives of General Psychiatry*, *4*(6), 561–571.

Eysenck, H. J., & Eysenck, S. B. G. (1975). *Manual of the Eysenck Personality Questionnaire*. London, UK: Hodder and Stoughton.

Eysenck, S. B. G., Eysenck, H. J., & Barrett, P. (1985). A revised version of the psychoticism scale. *Personality and Individual Differences*, *6*(1), 21–29.

Fox, J., & Weisberg, S. (2011). *An R companion to applied regression* (2nd ed.). Thousand Oaks, CA: Sage.

R Core Team. (2015). R: A language and environment for statistical computing [Computer software manual]. Vienna, Austria. Retrieved from `http://www.R-project.org` (ISBN 3-900051-07-0)

Revelle, W. (2015). psych: Procedures for psychological, psychometric, and personality research [Computer software manual]. Retrieved from `http://CRAN.R-project.org/package=psych` (R package version 1.5.6)

Table 1

Three EPI dimensions and associated traits

Psychoticism	Extraversion	Neuroticism
Aggressive	Sociable	Anxious
Assertive	Irresponsible	Depressed
Egocentric	Dominant	Guilt feelings
Unsympathetic	Lack of reflection	Low self-esteem
Manipulative	Sensation-seeking	Tense
Achievement-oriented	Impulsive	Moody
Dogmatic	Risk-taking	Hypochondriac
Masculine	Expressive	Lack of autonomy
Tough-minded	Active	Obsessive

Table 2

Regression Fit for BDI and EPI Neuroticism

	Estimate	Std. Error	t value	Pr(>|t|)
(Intercept)	-0.3213	0.7307	-0.44	0.6606
epiNeur	0.6820	0.0635	10.74	0.0000

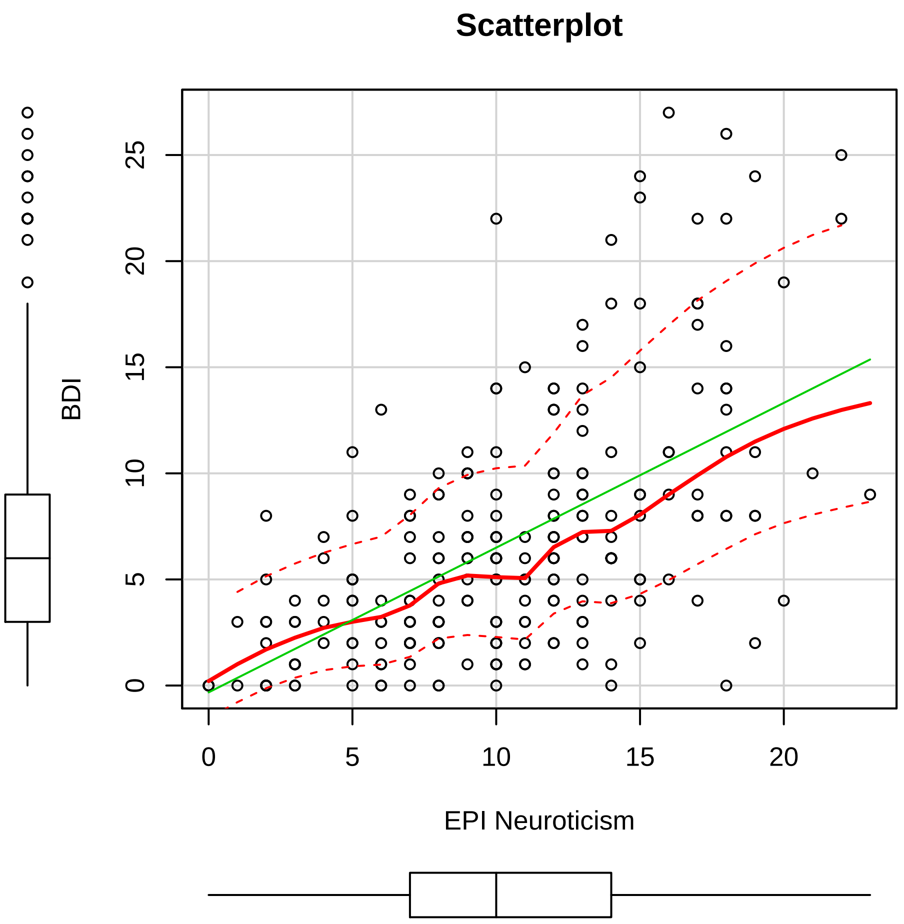

Figure 1. Scatterplot for EPI neuroticiscm and BDI including regression line.
